# Advances in Nutrition in Pediatric Gastroenterology

**DOI:** 10.3390/nu15092181

**Published:** 2023-05-04

**Authors:** Usha Krishnan, Andrew S. Day

**Affiliations:** 1 Paediatric Gastroenterology, Sydney Children’s Hospital, Sydney, NSW 2031, Australia; andrew.day@unsw.edu.au; 2 School Women’s and Children’s Health, University of New South Wales, Sydney, NSW 2052, Australia; 3 Department of Paediatrics, University of Otago Christchurch, Christchurch 8011, New Zealand

Chronic conditions affecting the gastrointestinal (GI) tract commonly impact nutrition adversely. This is especially relevant in children and adolescents with chronic GI conditions, where growth and development are key outcomes.

The nutritional impacts of chronic GI conditions in childhood include weight loss, or reduced weight gain, impaired linear growth, and delayed pubertal development. In addition, the malabsorption or reduced intake of micronutrients may lead to specific deficiencies, with potential further adverse effects. Iron deficiency can manifest as anemia but also impacts learning and development. Low levels of calcium and/or vitamin D can perturb bone health, with a consequent increased risk of fractures and the long-term development of osteopenia. Malnutrition in children can have a significant impact not only on health-related patient outcomes but also on the quality of life of children and their families. Examples of conditions that can have an impact on nutrition include inflammatory bowel disease, eosinophilic disorders, celiac disease, pancreatic disorders, chronic liver disease, intestinal failure, neurological disorders, cancer, and obesity. This Special Issue aimed to focus on the nutritional aspects of various GI conditions, with relevance to children and adolescence.

One condition that can present early in life with GI symptoms and impaired nutrition is food allergies, especially cow’s milk protein intolerance (CMPI). The Cow’s Milk-related Symptom Score (CoMiSS) was developed and validated as a tool to enhance awareness of the spectrum of symptoms related to CMPI and to quantify symptom thresholds. Vandenplas and colleagues [1] reported on an expert-opinion-driven reassessment of the CoMiSS tool, taking into account the data arising from 25 published reports using the tool. This re-evaluation recommended that the score indicating that symptoms are likely to be due to CMPI should be lowered from ≥12 to ≥10. In addition, the authors highlighted the need for further prospective assessment of the tool, along with its use in older infants. They also stressed that the CoMiSS should be seen as an awareness tool and not as a diagnostic test.

Various conditions, such as severe neurodevelopmental conditions, might lead to a need for ongoing assisted feeding in childhood. Dipasquale et al. [2] provided an overview of the role and application of tube feeding in children with neurodevelopmental conditions. Aspects included in this review were the methods of feed delivery, the type of nutrition provided, and the implications (such as upon quality of life). Key issues raised included the importance of considering steps to wean from tube feeding to oral feeding where feasible and safe. The role of blenderized tube feeds (BTFs) rather than an enteral formula was also discussed. Further to this aspect, Chandrasekar and authors [3] reported the outcomes of a prospective evaluation of BTFs compared to formula feeding in children who were tube-dependent. The use of BTFs resulted in several positive outcomes, including improved GI symptom scores. The children on BTFs also had lower stool calprotectin levels and a healthier as well as more diverse gut microbiome. Although the authors noted that the children who were fed with formula had better growth parameters than those fed solely with BTFs, this was not seen in those on partial BTFs, and even partial BTFs were enough to improve GI symptom scores.

Inflammatory bowel disease (IBD), which may present at any age during childhood, is commonly associated with malnutrition and micronutrient deficiency. Brown et al. [4] reported the findings of a case–control study involving children with IBD and their siblings without IBD living in the same household. The evaluation of dietary intakes and serum levels of key micronutrients demonstrated that many of these children did not meet the recommended daily intakes of several micronutrients. The children with IBD who were consuming additional enteral formula each day as part of their management plan had greater intakes of many micronutrients. Many of these children also had a low dietary fiber intake. Fiber intake is relevant to gut function as well as microbial functional changes, such as the production of short-chain fatty acids. Gerasimidis and colleagues [5] conducted in vitro assessments of the fermentation capacity of stools collected from individuals with IBD and controls without IBD. Whilst showing that there were differences in the patterns of the microbiome between the cases with IBD and controls, the data did not show any difference in fermentative capacity. This suggests that enhanced fiber intake may not lead to improvements in the functional outcome of the intestinal microbiome (but this study did not evaluate this further).

Another functional aspect of the intestinal microbiome is the ability to manage small fermentable sugars, known as the fermentable oligo-, di-, and mono-saccharides, and polyols (FODMAPs), which are inadequately digested in the small bowel. Rhys-Jones et al. [6] provided an overview of the role of a diet containing low amounts of FODMAPs in children. The authors emphasized the low number of studies assessing this dietary change in children, the importance of a skilled dietitian to safely guide the use of the diet, and the potential role of a FODMAP-gentle diet as a less restrictive intervention.

This collection of original research and review articles highlights some key aspects relevant to the impact of dietary interventions on children and adolescents. The assessment, monitoring, and optimization of outcomes might be seen as common threads running through these reports. In addition, however, these articles also serve to emphasize the gaps in our knowledge and understanding.
